# Expanding enhanced recovery protocols for cardiac surgery to include the patient voice: a scoping review protocol

**DOI:** 10.1186/s13643-020-01564-7

**Published:** 2021-01-11

**Authors:** Nebojša Oravec, Rakesh C. Arora, Brian Bjorklund, April Gregora, Caroline Monnin, Todd A. Duhamel, David E. Kent, Annette S. H. Schultz, Anna M. Chudyk

**Affiliations:** 1 Department of Surgery, Rady Faculty of Health Sciences, Max Rady College of Medicine, University of Manitoba, AE101-820 Sherbrook Street, Winnipeg, MB R3A 1R9 Canada; 2 Cardiac Sciences Program, CR 1005-St. Boniface Hospital, 369 Taché Avenue, Winnipeg, MB R2H 2A6 Canada; 3 Enhanced Recovery Protocols for Cardiac Surgery Patient Researcher Group, St. Boniface Hospital, 369 Taché Avenue, Winnipeg, MB R2H 2A6 Canada; 4 Neil John Maclean Health Sciences Library, University of Manitoba, 727 McDermot Avenue, Winnipeg, R3E 3P5 Canada; 5 Faculty of Kinesiology and Recreation Management, 208 Active Living Centre, University of Manitoba, Winnipeg, MB R3T 2N2 Canada; 6 Institute of Cardiovascular Sciences, St. Boniface General Hospital Albrechtsen Research Centre, 351 Taché Avenue, Winnipeg, MB R2H 2A6 Canada; 7 College of Nursing, Rady Faculty of Health Sciences, University of Manitoba, 89 Curry Place, Winnipeg, MB R3T 2 N2 Canada; 8 Health Services & Structural Determinants of Health Research, St. Boniface Research Centre, Winnipeg, Canada; 9 Department of Family Medicine, Rady Faculty of Health Sciences, University of Manitoba, 454-6 - 753 McDermot Avenue, Winnipeg, MB R3E 0 T6 Canada

**Keywords:** Cardiac surgery, Enhanced recovery, ERAS, Patient engagement, Caregiver, Scoping review, Protocol

## Abstract

**Background:**

Cardiac surgery is becoming increasingly common in older, more vulnerable adults. A focus on timely and complete medical and functional recovery has led to the development of enhanced recovery protocols (ERPs) for a number of surgical procedures and subspecialties, including cardiac surgery (ERAS® Cardiac). An element that is often overlooked in the development and implementation of ERPs is the involvement of key stakeholder groups, including surgery patients and caregivers (e.g., family and/or friends). The aim of this study is to describe a protocol for a scoping review of cardiac patient and caregiver preferences and outcomes relevant to cardiac surgery ERPs.

**Methods:**

Using Arksey and O’Malley’s et al six-stage framework for scoping review methodologies with adaptions from Levac et al. (Represent Interv: 1–18, 2012), a scoping review of existing literature describing patient- and caregiver-identified preferences and outcomes as they relate to care received in the perioperative period of cardiac surgery will be undertaken. The search for relevant articles will be conducted using electronic databases (i.e., the Cochrane Library, Medline, PsycINFO, Scopus, and Embase), as well as through a search of the grey literature (e.g., CPG Infobase, Heart and Stroke Foundation, ProQuest Theses and Dissertations, Google Advanced, and Prospero). Published and unpublished full-text articles written in English, published after the year 2000, and that relate to the research question will be included. Central to the design of this scoping review is our collaboration with two patient partners who possess lived experience as cardiac surgery patients.

**Discussion:**

This review will identify strategies that can be integrated into ERPs for cardiac surgery which align with patient- and caregiver-defined values. Broadly, it is our goal to demonstrate the added value of patient engagement in research to aid in the success of system change processes.

**Supplementary Information:**

The online version contains supplementary material available at 10.1186/s13643-020-01564-7.

## Background

Heart disease remains the leading cause of death around the world, accounting for an annual loss of life of 8.9 million people in 2015 [1]. Advances in perioperative care (i.e., care before, during, and after surgery) have contributed to ongoing reductions in mortality and complication rates following cardiac surgery in increasingly older, vulnerable adults [2–4]. While these “hard” outcomes are of significant value, it is becoming increasingly evident that attention also needs to be given to enhancing recovery of patients after surgery through, for example, a focus on patient-centered outcomes (i.e., functional capacity and rehospitalization rates) and health-related quality of life [5]. Patients typically spend between 4 and 7 days in hospital following a non-complicated cardiac surgery procedure, during which time they are monitored for postoperative infections, cardiac dysrhythmias, renal dysfunction, delirium, and other complications [6, 7]. Thus, the perioperative period represents a critical point of intervention to implement strategies aimed at enhancing patient recovery.

In 2019, an enhanced recovery protocol (ERP) was established for patients undergoing cardiac surgery (ERAS® Cardiac) [8]. It describes a 22-point plan to promote early recovery and return to normal activities, as well as reduce complications, perioperative mortality, and length of hospital stay [9, 10]. The protocol was developed by representatives from the groups of doctors that carry out cardiac surgery or oversee the care of cardiac surgery patients (i.e., cardiac surgeons, anesthesiologists, and intensivists) and followed the 2011 Institute of Medicine *Standards for Developing Trustworthy Clinical Practice* Guidelines [11]. An area that could be viewed for improvement in the development of the protocol was the lack of central involvement from patients and their caregivers, a key stakeholder group. Notably, of the 20 current published ERP guidance documents (across many surgical subspecialties), only one involved caregivers as partners in the process used to arrive at the provided recommendations [12].

Patients and caregivers have an expertise that stems from their lived experience of a health issue that can provide unique insights into the design and conduct of research, including the development of clinical practice guidelines [13, 14]. By sharing their experiences and perspectives of the daily impact of cardiac disease and surgery, unmet needs, therapeutic burdens, balance of benefits and risk, and types of research questions most important to them, they can transform the research process from one that is directed *for* patients and their caregivers, to one that is now informed and/or directed *by* them [15]. This study takes place within the context of a cardiac surgery program at a Canadian tertiary care center that is focused on involvement of patients and their caregivers in the research processes that influence their care. It is the first of several phases of research which seeks to engage patients and caregivers as research partners in the implementation of the ERAS® Cardiac guidelines at the tertiary care center. The specific aims of this study are to present a protocol for a scoping review of patient and caregiver preferences and outcomes as they related to the perioperative period of cardiac surgery. The results of the scoping review will contribute to a more comprehensive understanding of patient and caregiver preferences and prioritized outcomes relevant to ERPs for cardiac surgery and thus support patient-centered care.

## Methods

Similar to other types of literature reviews, such as systematic reviews, scoping reviews use rigorous and transparent methods to comprehensively identify and analyze all the relevant literature that helps to answer a research question [16]. They do not, however, involve the formal evaluation of the quality of scientific evidence included in the review [17]. Since the over-arching goal of scoping reviews is to develop a broad understanding, including identifying gaps in knowledge within a research area, they are useful when the existing literature has not been extensively reviewed or is heterogeneous (e.g., has mixed approaches to studying the topic of interest) [18]. For our research question, a scoping review is more appropriate than other methods of literature review because the topic (i.e., patient and caregiver preferences and outcomes as they related to the perioperative period of cardiac surgery) is broad, of a qualitative nature, and has not been extensively studied in this patient population.

### Patient engagement

Patient engagement in research involves meaningful and active collaboration between researchers and patients (and their caregivers) throughout the phases of a research project, including evaluation, planning, data collection and analysis, and knowledge translation [17]. Patients who engage in these collaborations may be referred to as patient partners. Two individuals (AG and BB) who received cardiac surgical care at our institution were engaged as patient partners in the development of this scoping review protocol. They collaborated on the design of the entire protocol and will continue to collaborate on the research activities (i.e., conduct, analysis, and knowledge translation) of the actual scoping review.

### Team composition and setting

Our research team is comprised of nine individuals and encompasses a broad range of expertise. Central to our study is the input of two patient partners (AG and BB) with lived experience of undergoing and recovering from cardiac surgery. The team also includes two researchers (AC and AS) with expertise in patient engagement in research, one cardiac surgeon/intensivist centrally involved in the development of the ERAS® Cardiac guidelines (RA), and a researcher (TD) with a program focused on cardiovascular health. CM is an expert librarian at the Neil John Maclean Health Sciences Library at the University of Manitoba. DK is a research coordinator within our institution’s Cardiac Sciences Program. The first author (NO) is a medical student in the Max Rady College of Medicine at the University of Manitoba.

### Reporting guidelines

The planning and documentation of our scoping review protocol follows the Preferred Reporting Items for Systematic Review and Meta-Analysis Protocols (PRISMA-P) checklist (Additional file 1) and the PRISMA extension for scoping reviews (PRISMA-ScR; Additional file 2). The reporting of patient engagement activities that we undertook as part of the development of this protocol are reported in accordance with the Guidance for Reporting Involvement of Patients and the Public 2 (GRIPP2) short-form (Additional file 3) [19]. This study was not prospectively registered in any database of literature reviews (e.g., PROSPERO) as it is not applicable.

### Scoping review methodology

This scoping review’s protocol follows Arksey and O’Malley’s six-stage framework for scoping review methodologies [17], with revisions from Levac et al. [20]. It describes six stages of research which we will use to guide our scoping review and expand upon in the sections that follow: (1) identifying the research question; (2) identifying relevant studies; (3) study selection; (4) charting the data; (5) collating, summarizing, and reporting the results; and (6) consultation.

### Stage 1: Identifying the research question

The first stage of a scoping review seeks to identify a research question that is broad enough to encompass many different ideas but narrow in its definition of the research concept, target population, and outcomes of interest. The rationale for the scoping review and its intended outcome should help define the research question [21].

#### The research question

We set out to conduct this scoping review because the input of cardiac surgery patients and caregivers is missing from the process used to arrive at current ERAS® Cardiac guidelines. Intended outcomes include patient- and caregiver-identified preferences and outcomes as they relate to perioperative care in cardiac surgery and the lifelong impact of cardiac surgery on the patient. Thus, this review seeks to answer the main research question as follows:

*What does the existing literature say about patient- and caregiver-identified preferences and outcomes as they relate to care received in the perioperative period of cardiac surgery and the lifelong impact of cardiac surgery on the patient?*

The main concepts within this research question are presented in accordance with the population, context, concept (PCC) framework (Table 1).

**Table 1:** Population, concept, context (PCC) framework for determining the eligibility of the research question

Criteria	Determinants
Population	**Adult cardiac surgery patients** and their **caregivers**. **Adult cardiac surgery patient** is defined as a person over the age of 18 who has undergone a surgical operation of the heart or great vessels (thoracic aorta, superior/inferior vena cava, pulmonary arteries/veins). **Caregiver** refers to those persons with interest in the patient’s wellbeing who are *not remunerated* for their role in the patient’s life.
Concept	(a) **Outcomes** that are important to patients and caregivers. ** Outcome** refers to both acute perioperative status and the lifelong impact of cardiac surgery on the patient. (b) Patient and caregiver **preferences**. **Preference** refers to what is important and/or prioritized in relation to the patient’s life after cardiac surgery.
Context	**Perioperative period of cardiac surgery**, including care received through enhanced recovery protocols.

### Stage 2: Identifying relevant studies

The second stage of a scoping review involves laying out a comprehensive plan of how we will (i) search and (ii) screen articles for inclusion in our review. The search strategy must balance comprehensiveness with feasibility. In the case of our scoping review, this requires acknowledging the limitations of time and personnel to ensure that the results of the search are manageable in terms of size of the final dataset and its interpretation.

#### Search methods

The literature search will be conducted using the following electronic databases: the Cochrane Library; Medline (Ovid); PsycINFO (Ovid); Scopus; and Embase (Ovid). The grey literature search will include searches for guidelines, policies, protocols, reports, and theses from a variety of different resources including CPG Infobase, Heart and Stroke Foundation, ProQuest Theses and Dissertations, Google Advanced, and Prospero. This formal search will be supplemented by reference checking. An expert librarian (CM) will work with the core research team (NO, AG, BB, AC) to develop and execute the search strategy in Medline. The librarian will adapt the Medline search to the other databases. The search strategy will be peer-reviewed by another librarian using the Peer Review of Electronic Search Strategy (PRESS) checklist [22]. All resources will be exported to EndNote (version x8), and the screening process will be completed in the free systematic review management software, Rayyan. A sample search strategy is presented in Additional file 4.

#### Inclusion and exclusion criteria

An article will be included if it meets all of the following criteria: (i) investigates the outcomes and/or preferences defined in stage 1, (ii) focuses on adult (aged ≥ 18 years) patients undergoing elective or emergent cardiac surgery and/or their informal or unpaid caregivers, and (iii) relates to care received during the perioperative period of cardiac surgery (including care received through ERPs). Both published and unpublished articles (e.g., reports, government and other agency documents, guidelines, policies) will be considered for inclusion.

Due to limited resources, articles that are not written in English will be excluded. In keeping with the design of the systematic reviews and meta-analyses used to develop the ERAS® Cardiac guidelines, articles published before the year 2000 will also be excluded. We will additionally exclude articles that are unavailable as full texts. Inclusion and exclusion criteria may be modified as the scoping review progresses based on increasing familiarity with the literature.

### Stage 3: Study selection

A four-step process will be used for study selection:

(i) Titles of articles retrieved through execution of the search strategy in each database will be screened by a single reviewer (NO). This reviewer will apply the inclusion/exclusion criteria and record whether the article is included or the primary reason for exclusion.
(ii) Inclusion/exclusion criteria will be applied to the abstracts of relevant articles identified in step 1 by two reviewers (NO and AC). Reviewers will meet at the beginning and end of the abstract screening process to discuss challenges and uncertainties related to study selection and refine the search strategy as needed. A third reviewer (RA) will resolve disagreements about inclusion/exclusion as necessary.
(iii) The protocol described in step 2 will be applied to screen the eligibility of the full texts of articles included at the end of step 2 by two reviewers (NO and AC).
(iv) The final set of included articles will also contain those that meet inclusion criteria and are identified by searching reference lists of the articles and other literature included at the end of stage 3.

**Fig. 1:**
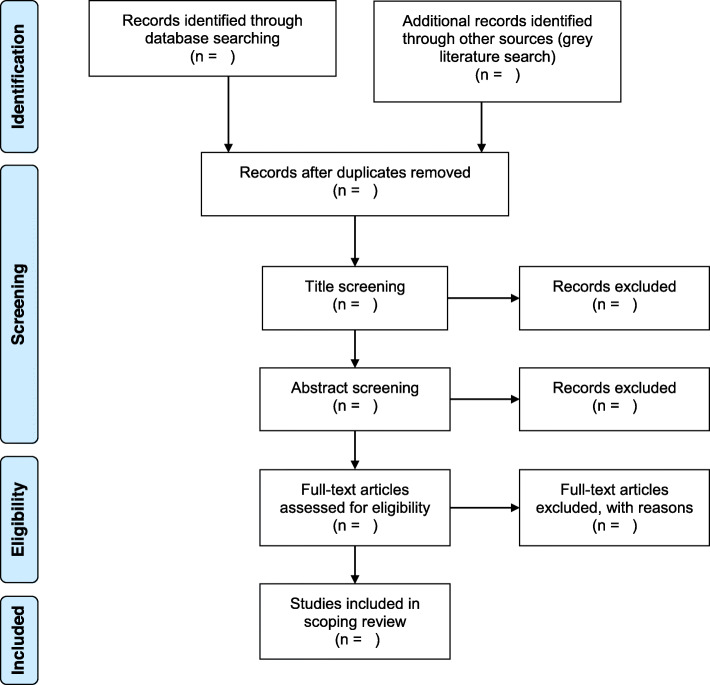
PRISMA flowchart for study selection

The study selection stage is an iterative process which may involve a refinement of the search strategy and multiple reviews of the complete citation list. Prior to the start of stages 1 and 2, inter-rater reliability will be established between the two reviewers (NO and AC) responsible for study selection through screening the titles and abstracts (respectively) of a random subset of 200 articles. The workflow for study selection is summarized in a PRISMA flowchart (Fig. 1). This figure will be used to report the number of citations at each stage of screening and selection.

### Stage 4: Charting the data

The goal of stage 4 is to collect information from individual articles which will aid in the development of a broad interpretation of the current literature as it relates to the research question. “Charting” in a scoping review is analogous to the “data extraction” process involved in a systematic review. It is defined as “a technique for synthesizing and interpreting qualitative data by sifting, charting, and sorting material according to key issues and themes” [23].

The data charting form was collectively developed by the core research team and includes both general information about the study and specific information relating to the research question (Table 2). Items on the charting form directly pertaining to the research question were selected for their ability to become synthesized as overarching themes or trends. The charting form also includes identified preferences (including priorities) if employed, along with the corresponding outcome (perceived or objective). Finally, specific charting items were included to capture whether studies engaged stakeholders (e.g., patients and/or caregivers) as research partners as well as details about the engagement process (i.e., the method and time point of stakeholder consultation and whether the stakeholders represent a procedure, disease, or setting subset of all cardiac surgery patients). No assumptions or simplifications have been made regarding any of the variables on the charting form. The only charting variable which requires reviewer interpretation is the “Limitations/risk of bias” field under the “Study characteristics” subheading.

**Table 2:** Proposed variables to be charted, by category

Category	Variable
**Publication characteristics**	Title
	Year of publication
	Journal/source (if unpublished)
	Published (yes/no)
**Author characteristics**	Surnames
	Countries
	Discipline; point of view; “lens”
**Study characteristics**	Design
	Population a. Procedure/operation subgroup (if applicable; e.g., emergent vs. elective) b. Disease subgroup (if applicable) c. Setting
	Primary outcome(s)
	Outcome measures
	Limitations/risk of bias
**Stakeholder characteristics**	Who was the stakeholder (patient/caregiver/both/other)?
	Total number of stakeholders
	Method of engagement (if applicable)
	Engagement time point (i.e., at which stage(s) of the research design? If applicable)
**Patient- or caregiver-focused results**	Patient- or caregiver-identified preferences (including priorities) a. What is the preference? b. Before/during/after/long after surgery? c. Measure(s) and associated outcome(s)
	Patient- or caregiver-prioritized outcome(s) a. What is the outcome? b. Before/during/after/long after surgery? c. Measure(s)

The initial data charting form will be calibrated among reviewers by comparing the results of independent data extraction from the first 5–10 studies, followed by a discussion regarding the consistency of the approach with respect to the research question and purpose [24, 25]. Data will be charted in duplicate (NO and AC), with data records managed and stored using Microsoft Excel. Inconsistencies will be resolved by a third reviewer (RA). It may not always be possible to obtain every charting item for each study. Studies will be excluded if the reviewers find that an insufficient amount of information can be obtained during this process. Like stages 2 and 3, charting the data is an iterative process that may require modifications to the data charting form based on increasing familiarity with included studies.

### Stage 5: Collating, summarizing, and reporting the results

In stage 5, we will construct a narrative account of included articles based on an inductive analytical framework or thematic construction. This stage will involve three steps: (i) descriptive numerical and/or qualitative thematic analysis, (ii) reporting our results and generating the intended outcome, and (iii) considering the meaning of the results of the review and their implications on research, policy, and medical practice [17]. Based on our a priori discussions with patient partners and the broader research team, we anticipate thematic construction to be the most appropriate data analysis method, and we propose a number of thematic categories of potential interest (Table 3). This process will involve the entire research team in a multi-step qualitative analysis that consists of five major steps as outlined in Table 4. Briefly, the data extraction step will involve transcription of individual statements of preference and prioritized outcome from the final set of included articles. Next, the familiarization step will require a thorough read of these extracted preferences by patient partners and the broader research team. Following idea generation and thematic grouping, the statements of preference or prioritized outcome will be uploaded to NVivo, and codes relating to theme, perioperative time, and other potentially relevant variables will be applied (i.e., coding). Once the dataset has been coded in its entirety, similar statements will be grouped into sub-themes, and this list will be presented and modified by the research team (i.e., thematic review). The final themes and sub-themes will be arrived at by consensus discussion with the research team to ensure their completeness and relevance, and to ensure that there are no duplicate or overlapping ideas (i.e., final definitions).

**Table 3:** Potential themes by result category

Patient preferences	Caregiver preferences	Prioritized outcomes
Education	Education	Function
Outpatient support	Emotional support	Reduced complications
Psychosocial support	Home care supports	Return to activities
Rehabilitation	Respite services	Survival
Tailored information	Communication	Quality of life

**Table 4:** Summary of tasks for thematic analysis

Task	Description
**Extraction**	Statements of patient and caregiver preference, and prioritized outcomes will be extracted from the final set of included articles.
**Familiarization**	All members of the research team will read the list of included statements and will group common themes and generate ideas for thematic analysis.
**Coding**	Statements of preference or prioritized outcome will be uploaded to NVivo. Codes relating to theme, perioperative timepoint, and other variables of interest will be applied.
**Thematic review**	Similar statements within each theme will be grouped into sub-themes, and this list will be presented to the research team for review, discussion, and modification.
**Final definitions**	Final themes and sub-themes will be arrived at by consensus discussion to ensure completeness and relevance, and to eliminate duplicate or overlapping ideas.

Although we are prepared for a thorough thematic analysis, the research team will work together to determine the most appropriate way to analyze and report the findings of the scoping review once the literature has been extensively reviewed. The rationale for selection of an analytic framework instead of a thematic analysis will consider whether the review findings primarily quantify preferences and outcomes, wherein a quantitative analytical framework may be more appropriate, or if they are primarily qualitative, in which case we would proceed with thematic analysis.

Regular meetings and consensus discussions will aim to eliminate biases and strive to achieve a mutual interpretation of the review findings. This stage will comprise the “Results” and “Discussion” sections of the final manuscript. Evidence will be presented in a variety of formats (e.g., narrative, visual, table) depending on the type of data analysis performed.

### Stage 6: Consultation

Consultation with persons who have a defined interest or life experience relating to a research outcome is an essential stage of the scoping review. This is especially true in our review of patient- and caregiver-identified preferences and outcomes that will be applied to the re-development of clinical practice guidelines. The goal of consultation is to recognize lived experience as a legitimate form of knowledge that provides insights distinct from the knowledge of academics and health professionals. It also aims to reaffirm the position of the stakeholder at the center of the research focus. In the context of medical research, involving the patient in the design, conduct, and knowledge translation of a research project ensures that the findings are relevant to the target population.

Our research team includes two patient partners (AG, BB) who are alumni of the Cardiac Surgery Program at St. Boniface Hospital, with experience as stakeholders on other research projects relating to cardiac surgery. A patient engagement grant has allowed AG and BB to work with the research team as collaborators rather than consultants from the planning stages of the scoping review and onwards. Specifically, AG and BB are members of the core research team, which meets bi-weekly to address all aspects relating to the scoping review’s development and conduct. At the outset of their membership on the research team, they collaborated on the co-development of terms of reference document to help establish their interests and responsibilities within the research team, as well as the intended working environment and nature of the relationship between patient partners and other researchers. This is a “living document” that may be revised as necessary throughout the research process. A summary of this document’s content is presented in Table 5.

**Table 5:** Content of the terms of reference document for patient engagement

Subsection	Key components
Project overview	A description of the research project, including background information and overarching goals.
The research team	Members of core working group and their positions.
Responsibilities and opportunities for patient researchers, patient researcher liaison/facilitator, and the broader research team	a. Patient researchers—to participate in the research process, communicate concerns to the research liaison, prepare for meetings by reviewing documents, etc. b. Patient researcher liaison/facilitator—to communicate regularly with the patient researchers, listen to and address concerns, ensure integration of patient researchers into the research team, etc. c. Research team—value lived experience as a form of knowledge, use knowledge appropriately and confidentially, maintain fair and structured relationships, be mindful of word choice in written materials.
Process (work plan)	A model for the process of patient engagement during biweekly meetings; a project timeline outlining milestones and frequency of full-team meetings.
Expected outcomes	Major project milestones, including formal publications, non-traditional knowledge sharing activities, future research projects, etc.
Our working environment	A description of the environment fostered by the research team, as guided by the principles within Strategy for Patient-Oriented Research’s Patient Engagement Framework (i.e., mutual respect, inclusiveness, co-building, and supports (safe space, educational supports, financial supports) [26].

The impact of patient partners on the research is being documented through meeting minutes and document revisions. In the scoping review’s first stage, their key contributions included (i) ensuring that the research question reflected patient-centered ideas surrounding the concept of enhanced recovery and (ii) that the expansion of priorities and outcomes within the PCC framework to also include the distant impact of cardiac surgery on the patient’s life, as opposed to a sole focus on the acute, in-hospital postoperative recovery period. In preparation for stage 2, patient partners also helped identify search terms, sources of grey literature, as well as preliminary inclusion/exclusion criteria. We anticipate a number of roles and potential impacts of patient partners through subsequent stages of scoping review. In stages 2–3, the search strategy and a summary result of each stage of study screening will be presented to the patient partners at bi-weekly meetings. At this time, patient partners will reflect on their lived experience to ensure that any amendments to the search strategy and inclusion/exclusion criteria support a patient-centered understanding of recovery. Similarly, in stage 4, finalization of the charting form will include the variables proposed by the patient partners. In stage 5, statements of preference and prioritized outcomes will be extracted from included articles and presented to the patient partners for initial thematic grouping and idea generation. The reviewers (NO and AC) will apply and modify this initial, patient-oriented thematic perspective to the entire dataset in NVivo. Modifications and alternate theme development will be presented to patient partners and modified accordingly.

In this study, the consultation stage will also involve a half-day workshop wherein the results of the scoping review will be shared with a group of patient and caregiver representatives. Patient partners (AG and BB) will not only help plan the workshop but also lead research presentations and act as small-group facilitators. This workshop will serve both as a knowledge translation activity and an additional source of patient/caregiver information that will be used to validate and aid in interpreting the results of the scoping review. This event will also likely result in a separate peer-reviewed publication and contribute to the development of informal resources for cardiac surgery patients and their caregivers.

The impact of our engagement with patient partners will be measured and evaluated through formal documentation including a self-report survey and qualitative interviews with all researchers at the conclusion of the study. We will assess the perceived impacts of patient engagement on the design and conduct of the scoping review. The work of patient engagement will also be disseminated in non-traditional ways (e.g., development of a website, educational material for cardiac surgery patients). Patient partners (AG and BB) will participate in the authorship and development of all manuscripts and knowledge translation products related to this research.

## Discussion

The proposed scoping review will aid in the identification of factors that patients and their caregivers consider as being important to their care before, during, and after cardiac surgery. It is our goal to use the results of this scoping review to inform the implementation of a patient-centered, cardiac-focused ERP at our institution and to identify strategies that patients themselves can use to influence their health and recovery.

In the development of this protocol, the aim of patient engagement was to increase the likelihood that the rationale, design, and findings of the scoping review agreed with the lived experiences of cardiac surgery patients. In all stages of protocol development, patient partners helped ensure that the process reflected values and experiences derived from their experience as cardiac surgery patients. Specifically, the patient partners were central in the development of the research question and the charting form. They emphasized the importance of collecting information concerned with long-term outcomes after surgery, including return to “normal life.” Recognizing the lack of peer-facilitated patient education in cardiac surgery, the patient partners were also interested in facilitating non-traditional dissemination of the results of the scoping review (e.g., development of patient resources, website). Additionally, the process of patient engagement encouraged other members of the research team to evaluate whether the study could be translated into accessible language that allowed patients themselves to assess whether the study accurately reflected their experiences. This process helped confirm that the research did in fact address factors that patients experience as valuable to their health and recovery.

The strengths of this protocol relate to its emphasis on patient engagement and its context within a larger research design aimed at implementing ERAS® Cardiac guidelines with a patient-centered focus. A significant advantage of the design is in the composition of the research team, which represents a broad range of knowledge and includes two patient partners who have experienced cardiac surgical care. The research design also allows for the results of the scoping review to be used to directly influence patient care, within subsequent patient-centered ERP implementation phases. That said, our scoping review protocol is limited by the fact that it did not engage caregivers as research partners. Though absent from the current research team, these stakeholders’ perspectives will likely be captured through the findings of the scoping review and directly through subsequent phases of the long-term research plan. Although thematic analysis is appropriate for synthesizing the results of large, mostly qualitative datasets, it is subject to the biases of those who construct the themes. Therefore, such a method can be considered feasible only when biases can be addressed and attempts can be made to minimize them. For our analysis, it will be important to involve patient partners at the early stages of data analysis, including an initial idea-generating and thematic grouping activity for the extracted statements of preference and prioritized outcomes, before other members of the research team introduce their biased perspectives as health care providers or academics. We also attempt to minimize potential bias in the interpretation and reporting of our findings by obtaining feedback on the review findings from patients and caregivers at the half-day consultation workshop. During this activity, stakeholders will be able to share whether they find the thematic groupings applicable, relevant, and consistent with their experience as cardiac surgery patients or caregivers. Subsequently, the analysis will be modified to reflect the patient and caregiver perspective.

The outcomes of this scoping review will be published in a peer-reviewed journal, presented at conferences and to staff within the Cardiac Sciences Program at St. Boniface Hospital, and used to develop patient resources. We are also developing a website (http://www.patientengagementinresearch.ca) to disseminate this and other research we carry out in partnership with patients to non-academic audiences. We hope to demonstrate the value-added aspects of a patient-centered research design through the success of this scoping review in identifying patient- and caregiver-identified preferences and outcomes that will be used to influence patient care. Outcomes of our scoping review will advance guideline development beyond addressing clinician-focused enhanced recovery interventions. Review findings will shed light on patient and caregiver preferences and outcomes that may be self-initiated strategies. Gaining insight into these patient and caregiver strategies may be empowering for patients to confidently and actively influence his or her own health.

## Supplementary Information

**Additional file 1.** PRISMA-P checklist.

**Additional file 2.** PRISMA-ScR checklist.

**Additional file 3.** GRIPP2 checklist.

**Additional file 4.** Preliminary search strategy for Medline.

## Data Availability

All data generated or analyzed in this study will be included in the published scoping review article. Other resources can be made available upon request.

## Abbreviations

ERP: Enhanced recovery protocol
ERAS® Cardiac: Enhanced recovery after cardiac surgery
PRISMA-P: Preferred reporting items for systematic review and meta-analysis protocols
PRSIMA-ScR: Preferred reporting items for systematic review and meta-analysis scoping reviews
GRIPP2: Guidance for Reporting Involvement of Patients and the Public 2
PCC: Population, concept, context
PRESS: Peer Review of Electronic Search Strategy
PRISMA: Preferred reporting items for systematic review and meta-analysis
