# Supplementary material for: Expanding enhanced recovery protocols for cardiac surgery to include the patient voice: a scoping review protocol
Source: Syst Rev. 2021 Jan 11;10:22. doi: 10.1186/s13643-020-01564-7 (PMC7798193; doi:10.1186/s13643-020-01564-7)
Supplement: Supplementary file 3 — Additional file 3. GRIPP2 checklist. [file 13643_2020_1564_MOESM3_ESM.docx]

| Section and topic | Item | Reported on page No |
| --- | --- | --- |
| 1: Aim | Report the aim of PPI in the study | 7 |
| 2: Methods | Provide a clear description of the methods used for PPI in the study, including both positive and negative outcomes | 9, 15-19 |
| 4: Discussion and conclusions | Outcomes—comment on the extent to which PPI influenced the study overall. Describe positive and negative effects | 17-18 |
| 5: Reflections/critical perspective | Comment critically on the study, reflecting on the things that went well and those that did not, so others can learn from the experience | 17-18, 20-21 |

Additional file 3. GRIPP2 short form.

PPI = patient and public involvement
