# Supplementary material for: Expanding enhanced recovery protocols for cardiac surgery to include the patient voice: a scoping review protocol
Source: Syst Rev. 2021 Jan 11;10:22. doi: 10.1186/s13643-020-01564-7 (PMC7798193; doi:10.1186/s13643-020-01564-7)
Supplement: Supplementary file 4 — Additional file 4. Preliminary search strategy for Medline. [file 13643_2020_1564_MOESM4_ESM.docx]

| Search number | Search terms |
| --- | --- |
| **Search terms relating to the study population** | |
| 1 | Thoracic Surgery/ or Cardiovascular Surgical Procedures/ or exp Cardiac Surgical Procedures/ |
| 2 | ((heart or cardio$ or cardiac$ or thoracic$ or arter$ or aort$ or mitra-valve or mitra or tricuspid-valve or tricuspid or pulmonary-valve or valve or heart-lung or maze or ross or david or bentall) adj1 (operat$ or surg$ or procedur$ or repair$ or replacement$ or massag$ or transplant$ or grafting$ or by-pass$ or bypass$)).tw,kw. |
| 3 | (annuloplast$ or cardiomyoplast$ or cardioplegia$ or CABG or aortic-valve-implant$ or heart-valve-prothesis-implant$ or myocardial-revascula?ation$ or angioplast$ or coronary-balloon$ or coronary-atherectom$ or rotational-atherectom$ or mammary-artery-anastomos$ or pericardial-window$ or pericardiostom$ or Pericardiectom$ or Pericardiocentes$ or TAVR or TAVI or aortic-root or left-venticular-assist-device$ or LVAD or extracorporeal-membrane-oxygenation or ECMO or myectom$ or myotom$ or cardiac-tumo?r or cardiac-neoplasm$ or atrial-my?xoma or aortic-aneurysm or root-aneurysm or arch-aneurysm or thoracic-aneurysm or thoraco-abdominal-aneurysm or aortic-dissection).tw,kw. |
| 4 | or/ 1-3 |
| **Search terms relating to the research context** | |
| 5 | exp Perioperative Period/ or exp perioperative care/ or intraoperative care/ or postoperative care/ |
| 6 | (perioperat$ or postoperat$ or preoperat$ or intraoperat$ or ((peri or post or pre or intra) adj1 (operat$ or surg$ or procedur$)) or long-term-outcome? or longterm-outcome?).tw,kw. |
| 7 | (ERAS or early-recover$ or (enhanc$ adj3 (recover$ or function$ or protocol$))).tw,kw. |
| 8 | or/ 5-7 |
| **Search terms that to the research concept** | |
| 9 | ((research$ or setting$ or patient?) adj2 (priorit$ or partner$)).tw,kw |
| 10 | (patient-centered or patient-centred or PCC or patient-focused or patientcentred).tw,kw. |
| 11 | ((relation$ or communication$) adj3 (famil$ or patient? or care-giver? or caregiver? or parent$ or carer? or spouse?)).tw,kw. |
| 12 | Patient participation/ or "Patient Acceptance of Health Care"/ or exp Attitude to Health/ or patient satisfaction/ or patient preference/ or cooperative behavior/ or self efficacy/ or exp Adaptation, Psychological/ or exp health education/ or health knowledge, attitudes, practice/ or *"Quality of Life"/ or "Quality of Life"/px or Personal Autonomy/ or self concept/ or consumer advocacy/ or freedom/ or Needs Assessment/ or Patient Advocacy/ or Attitude to Death/ or exp Patient-Centered Care/ or Nurse-Patient Relations/ or Physician-Patient Relations/ or Researcher-Subject Relations/ or Self Care/ or narration/ or patient reported outcome measure/ or narrative medicine/ or professional-family relations/ or professional-patient relations/ |
| 13 | ((patient or consumer$ or public? or famil$ or care-giver$ or caregiver$ or parent$ or carer$ or spous$) adj3 (participat$ or decisi$ or decid$ or derid$ or involve$ or interest? or empower$ or satisfact$ or engag$ or education$ or attitude? or desir$ or perspective? or activation or view$)).tw,kw. (215493) |
| 14 | (patient$ adj3 (attitude? or preference?)).tw,kw. |
| 15 | ((self$ adj1 (perception? or concept? or efficac$)) or selfperception or selfconcept or selfefficac$).tw,kw. |
| 16 | (informed-choice? or shared-decision-making or expert-patient? or patient-expert or stakeholder-engagement? or stake-holder-engagement? or patient-outcome-assessment? or patient-reported-outcome? or PROM?).tw,kw. |
| 17 | (Quality-of-life or QOL).ti. |
| 18 | Return to work/ or (return adj2 (work or normal$ or life or function or social or activit$)).tw,kw. |
| 19 | or/ 9-18 |
| **Combined PCC search** | |
| 20 | 4 and 8 and 19 |
| 21 | limit 20 to English language |
| 22 | 21 not ((exp infant/ or exp child/ or adolescent/) not exp adult/) |
| 23 | limit 22 to yr=”2000-Current” |
